# Multinuclei Occurred Under Cryopreservation and Enhanced the Pathogenicity of *Melampsora larici-populina*

**DOI:** 10.3389/fmicb.2021.650902

**Published:** 2021-06-25

**Authors:** Wei Zheng, Zijia Peng, Shaobing Peng, Zhongdong Yu, Zhimin Cao

**Affiliations:** College of Forestry, Northwest A&F University, Yangling, China

**Keywords:** Melampsora larici-populina, multinucleation, transcriptomics, pathogenicity variation, adaptation, cryopreservation

## Abstract

*Melampsora larici-populina* is a macrocyclic rust, and the haploid stage with two nuclei and the diploid of mononuclear sequentially occur annually. During the preservation of dry urediniospores at −80°C, we found that one isolate, ΔTs_06_, was different from the usual wild-type isolate Ts_06_ at −20°C because it has mixed polykaryotic urediniospores. However, the other spores, including the 0, I, III, and IV stages of a life cycle, were the same as Ts_06_. After five generations of successive inoculation and harvest of urediniospores from the compatible host *Populus purdomii*, the isolate ΔTs_06_ steadily maintained more than 20% multiple nucleus spores. To test the pathogenesis variation of ΔTs_06_, an assay of host poplars was applied to evaluate the differences between ΔTs_06_ and Ts_06_. After ΔTs_06_ and Ts_06_ inoculation, leaves of *P. purdomii* were used to detect the expression of small secreted proteins (SSPs) and fungal biomasses using quantitative real-time PCR (qRT-PCR) and trypan blue staining. ΔTs_06_ displayed stronger expression of five SSPs and had a shorter latent period, a higher density of uredinia, and higher DNA mass. A transcriptomic comparison between ΔTs_06_ and Ts_06_ revealed that 3,224 were differentially expressed genes (DEGs), 55 of which were related to reactive oxygen species metabolism, the Mitogen-activated protein kinase (MAPK) signaling pathway, and the meiosis pathway. Ten genes in the mitotic and meiotic pathways and another two genes associated with the “response to DNA damage stimulus” all had an upward expression, which were detected by qRT-PCR in ΔTs_06_ during cryopreservation. Gas chromatography–mass spectrometry (GC-MS) confirmed that the amounts of hexadecanoic acid and octadecadienoic acid were much more in ΔTs_06_ than in Ts_06_. In addition, using spectrophotometry, hydrogen peroxide (H_2_O_2_) was also present in greater quantities in ΔTs_06_ compared with those found in Ts_06_. Increased fatty acids metabolism could prevent damage to urediniospores in super-low temperatures, but oxidant species that involved H_2_O_2_ may destroy tube proteins of mitosis and meiosis, which could cause abnormal nuclear division and lead to multinucleation, which has a different genotype. Therefore, the multinuclear isolate is different from the wild-type isolate in terms of phenotype and genotype; this multinucleation phenomenon in urediniospores improves the pathogenesis and environmental fitness of *M. larici-populina*.

## Introduction

**Figure 1:**
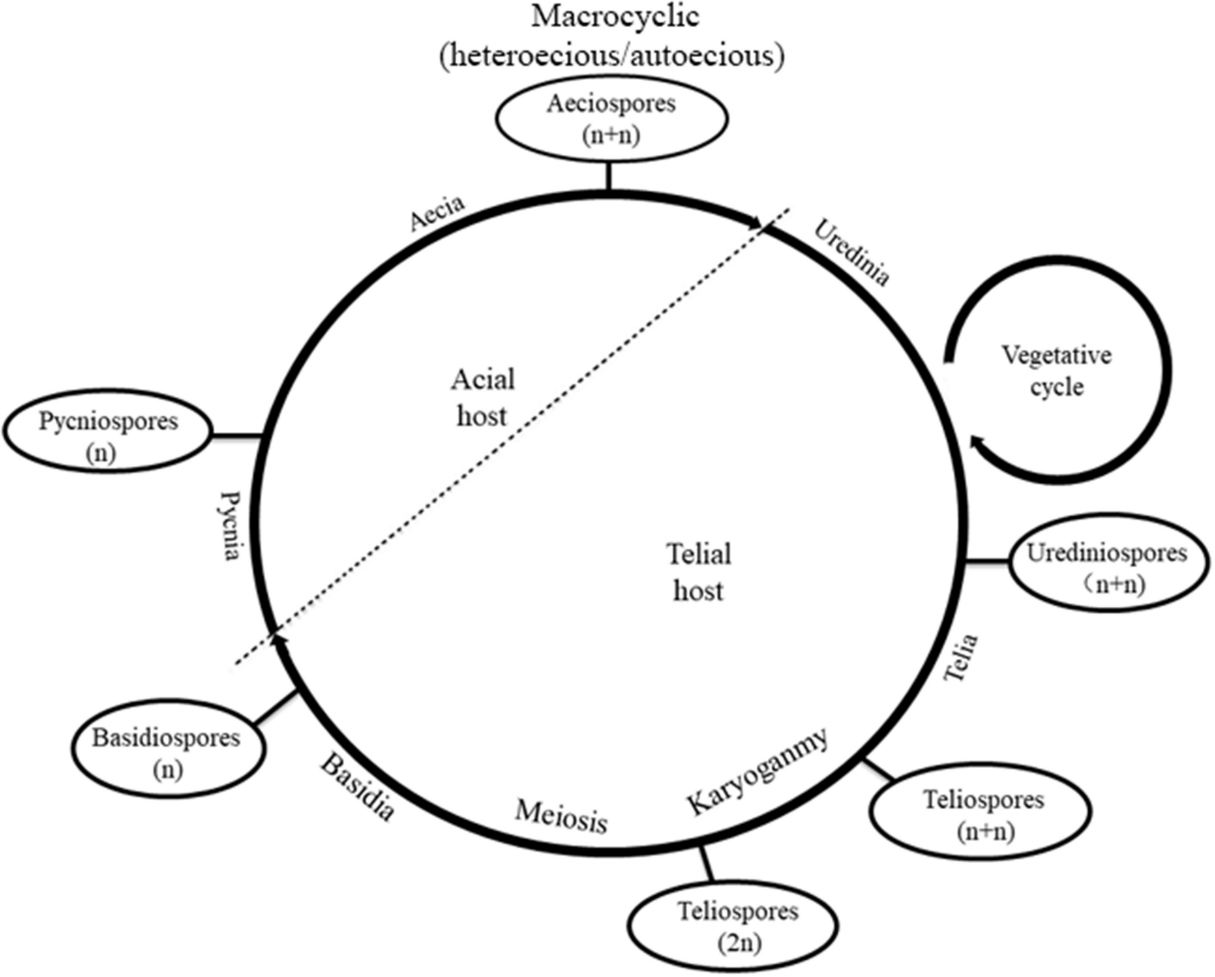
Life cycle of *Melampsora larici-populina*.

*Melampsora larici-populina* Kleb., an obligate biotrophic parasite, has caused heavy rust infections with disastrous consequences for the poplar industry (Steenackers et al., 1996; Newcombe, 1998). It is a macrocyclic rust that completes its life cycle on larch and poplar (Figure 1) and involves five types of spores: pycniospores (monokaryon), aeciospores (dikaryon), urediniospores (dikaryon), teliospores (diploid monokaryon), and basidiospores (monokaryon) (Lorrain et al., 2019). However, multinucleation has been observed in several rust fungi, including *M. larici-populina*, and the life cycle shows obvious diversity. For example, *Uromyces vignae* Barclay has a macrocyclic life cycle, but it completes the life cycle on a single species host, during which basidiospores develop into monopyrenous hyphae and urediniospores consist of eight or 16 nuclei. After the differentiation of haustorial mother cells, intercellular hyphae develop four to 12 nuclei, which gradually decrease over time. Primary haustorial mother cells have four or five nuclei but are rarely dikaryon (Heath et al., 1996). Septate hyphae of *Puccinia sorghi* were formed after the mitosis of one or two nuclei; hence, primary hyphae are trinucleate (Savile, 1939). The primary hyphae of *P. malvacearum* are divided into one anucleate cell and one binucleate cell, and the latter is divided into two monocytes by one septum (Allen, 1935). Multinucleation is very common during the infection process of *P. striiformis* f. sp. *tritici*, especially in germ tubes, intercellular hyphae, haustorial mother cells, and haustorial cells (Little and Manners, 1969; Kang et al., 2015). Germ tubes of the urediniospores of *M. larici-populina* fuse on the surface of the poplar leaves and form multinucleate cells (Yu et al., 2009), but the vegetative hyphae typically become dikaryotic hyphae when the multinucleate cells develop into infected hyphae. The dikaryon hyphae do not grow with clamp connections, and a sister nucleus in vegetative hyphae is copied in a “half-reserve” manner, with one disappearing or becoming retained and the other flowing into a new cell and remaining as a dikaryon through mitosis (Yu et al., 2017). When the basidiospores regerminate, binucleate secondary basidiospores are formed and can more easily infect the host (Yu et al., 2009).

Multinucleation can occur in the following ways: 1) germ tube fusion or hyphal fusion. Since 1989, fusant formation and fusion phenomena of germ tubes, appressoria, substomatal cavities, or hyphae have been observed in a variety of *Puccinia* species (Park and Wellings, 2012). Wilcoxon et al. (1958) discovered that the cytoplasm in germ tubes moved to the fusant, which had two to four nuclei. Taylor (1976) discovered that somatic recombination could produce a new race by albino strains of *P. striiformis* f. sp. *tritici*. The proportions of tri- and tetra-nucleate germ tubes in the new race were significantly higher than those in the original strains. 2) Nucleus mutation: factors for nuclear mutation include ion radiation, chemical action, temperature changes, and pH changes (Huang et al., 2009; Anderson et al., 2015). They can cause nuclear DNA damage and subsequently lead to abnormal mitosis and multinucleation (Maheshwari, 2005).

Multinucleation often leads to different cytological and biological traits, causing special life cycles and diverse environmental fitness, especially for macrocyclic rust fungi, whose haploid and diploid stages are strictly defined in their life cycle. Moreover, multinucleation can cause changes in phenotypes, including the latent period, uredinium density, uredinium diameter, and uredinium height in a range of hosts (Chandrasbekar and Heather, 1980). In the interaction between plants and pathogens, avirulence proteins are generally thought to be pathogenicity effectors that have positive roles. Some effectors are targeted to the host nucleus and may act as transcription factors (Lahaye and Bonas, 2001), whereas others are proteases that cleave specific cytoplasmic host proteins (Orth et al., 2000). Hacquard et al. (2012) found that parts of small secreted proteins (SSPs) in *M. larici-populina* are homologous to avirulence genes of *M. lini*. For example, Mlp124272 and Mlp124256 are homologous to AvrP4; Mlp37347 is homologous to AvrL567; Mlp124530 is homologous to AvrP123; and Mlp123932 is homologous to haustorially expressed secreted protein (HESP) HESP-327. Furthermore, specific labeling of Mlp37347 and Mlp123932 was detected at the periphery of haustoria. Mlp124272, Mlp124256, and Mlp124530 showed very strong labeling in the development of uredinia (Hacquard et al., 2012). SSPs that involved avirulence effectors were those important molecular associated with changing phenotypes. RNA sequencing (RNA-Seq), also known as transcriptome sequencing, is universally employed to analyze differential gene expression through deep sequencing during pathogen–host interactions (Wang et al., 2009). Over the past decade, RNA-Seq has gradually become an indispensable tool for analyzing differential gene expression (Stark et al., 2019). For example, Hacquard et al. (2013) revealed the karyogamy and meiosis processes before and after overwintering through transcriptome analysis of *M. larici-populina* telia.

Rust fungi are obligate parasites; hence, artificial culture technology is difficult to implement in biological research, including sporulation, mating, and subcellular structure under artificial synthesis media. Therefore, their spore propagulum is usually kept for research in dry conditions at −20°C for 1 year or at −80°C for more than 1 year. However, we found that multinucleation occurred in urediniospores of strain Ts_06_ stored at −80°C, and we obtained a mutant strain (ΔTs_06_) after purification and separation. The mutant strain, ΔTs_06_, was significantly different from Ts_06_ in host selection, pathogenicity, and other biological characteristics. Therefore, to investigate the genetic stability of mutated strains and the mechanism of multinucleation occurrence, we analyzed the stability of the life cycle of ΔTs_06_ using artificial inoculation on the compatible host *P. purdomii* for consecutive subcultures. The phenotype responses on different hosts and the expression of SSPs under real-time fluorescence quantitative analysis were used to determine the differences in pathogenicity between ΔTs_06_ and Ts_06._ The molecular mechanism of urediniospore multinucleation was explored using transcriptomics combined with real-time fluorescence quantitative PCR on nuclear division-related genes. The research results are of crucial theoretical significance in understanding the life history, pathogenicity variation, mechanism and environmental adaptability of such obligate parasites.

## Materials and Methods

### Materials

#### Strains

Mutant strain Ts_06_ (ΔTs_06_) at ultralow temperature (−80°C) and unmutated dikaryon strain Ts_06_. *P. purdomii* is the natural host of *M. larici-populina* collected in Qinling Mt., China. *P. purdomii*, the other four hosts (Table 1), and the aecial host of Chinese larch (*Larix principis-rupprechtii* Mayr.) were planted in greenhouse conditions at 25°C, 70%−80% relative humidity (RH), and 16 h photoperiod (2,500 lux at leaf level) for *in planta* experiments. They are healthy 1-year-old seedlings.

**Table 1:** Comparison of pathogenicity of ΔTs_06_ and Ts_06_.

		**Host**				
		***Populus purdomii***	***Populus deltoides***	***Populus szechuanica***	***Populus* × *euramericana* cv. Polska 15A**	***Populus* × *euramericana* cv. Robusta**
Latentperiod/days	ΔTs_06_	5	/	7	6~7	7
	Ts_06_	6	/	7~8	7	7~8
Sorus diameter/mm	ΔTs_06_	1.28 ± 0.12^a^	/	0.80 ± 0.07^a^	0.64 ± 0.06^a^	0.54 ± 0.07^a^
	Ts_06_	1.19 ± 0.10^a^	/	0.63 ± 0.04^a^	0.58 ± 0.08^a^	0.42 ± 0.05^a^
Sorus density/cm^−2^	ΔTs_06_	79.0 ± 2.60^a^	0^a^	13.0 ± 1.29^a^	52.0 ± 2.29^a^	6.0± 1.49^a^
	Ts_06_	63.0 ± 1.91^b^	0^a^	5.0 ± 0.93^b^	41.0 ± 1.78^b^	3.0 ± 0.55^b^
Sorus height/mm	ΔTs_06_	0.31 ± 0.03^a^	/	0.18 ± 0.04^a^	0.25 ± 0.02^a^	0.23 ± 0.02^a^
	Ts_06_	0.34 ± 0.03^a^	/	0.19 ± 0.02^a^	0.28 ± 0.02^a^	0.25 ± 0.02^a^

*Significance at p <0.05 is indicated by different letters in the table (Duncan's multiple range test)*.

### Nuclear Observation of the ΔTs_06_ Life Cycle

Nuclei were stained with DAPI (Sigma Chemical Company, St. Louis, MO, USA) and were dated under fluorescence microscope (Leica DM4000B, Leica Microsystems GmbH, Wetzlar, Germany). Urediniospores of ΔTs_06_ were prepared in spore suspensions at a concentration of 0.01 g/ml and were smeared on a 2% water agar medium for germination and on abaxial leaves of *P. purdomii* for inoculation as described in Zheng et al. (2019). Histological observation of intercellular hyphae, haustorial mother cells, and haustoria was implemented using a Hitachi HT-7700 TEM (Hitachi, Tokyo, Japan) (Kang and Buchenauer, 2000). Urediniospores of ΔTs_06_ were continuously subcultured on *P. purdomii* and harvested five times, and the proportion of polykaryotic urediniospores was counted in each harvest. Meanwhile, *P. purdomii* of 10 days post inoculation (dpi) were transferred to a chamber to induce teliospores at 4°C, and basidiospores developed according to the protocol outlined in Yu et al. (2009). Thereafter, basidiospores were inoculated on the needles of a larch, and then they were moved to the greenhouse until spermatia developed (Pernaci et al., 2014). The basidia, basidiospores, spermatia, and aeciospores were stained using DAPI and were observed and photographed using a Leica DM4000B microscope. The strain Ts_06_, used as a control, was observed simultaneously with ΔTs_06_.

### Determination of Pathogenicity and Fungal Biomass

*P. purdomii, P. deltoides, P. szechuanica, P*. × *euramericana* cv. Polska 15A, and *P*. × *euramericana* cv. Robusta were selected for pathogenesis tests (Pinon et al., 1987; Pinon and Frey, 1997). ΔTs_06_ and Ts_06_ at 0.01 g/ml of urediniospores were inoculated on each host using three biological replicates. The latent period (days), uredinia density (number per cm^2^), uredinia diameter (mm), and uredinia height (mm) were recorded after inoculation. To measure density, a 1-cm^2^ piece of paper was placed on the back of the leaves. The number of uredinia in at least 5-cm^2^ of paper per leaf was randomly counted to calculate the average density value. The uredinium diameter and height were observed and measured using an anatomical lens. All analyses were performed with SPSS 22.0 (Chicago, Chicago, IL, USA). The means of the densities were determined using Duncan's multiple range test at *p* <0.05. Simultaneously, leaves of *P. purdomii* were collected using a kit (Cat# BSC65S1B; BioFlux, San Francisco, CA, USA) at 0, 12, 24, 48, 96, and 168 h post inoculation (hpi) to obtain qualified RNA. The iScript™ cDNA kit was used for cDNA synthesis (Cat# 1708891; Bio-Rad Laboratories, Hercules, CA, USA). The SsoFast™ EvaGreen® Supermix kit was used for qPCR (Cat# 172-5201; Bio-Rad) with SSP primers, as shown in Table 2 (Hacquard et al., 2011; Vieira et al., 2011). Alpha-tubulin (*aTUB*) and elongation factor-1-alpha (*ELF1a*) of *M. larici-populina* were selected as reference genes, and amplification reaction conditions were the same as in Ye et al. (2018). The DNA of leaves during infection was extracted using a DNA extraction kit (Tiangen, Beijing, China). A species-specific ITS_1−F_/ITS_4−B_ of *M. larici-populina* was also employed to quantify the fungal biomass (Boyle et al., 2005). The gene expression was calculated using the 2^−ΔΔCt^ method of the dissolution curve (Livak and Schmittgen, 2001) in the quantitative fluorescent amplification. The two-tailed *t*-test was applied using the software GraphPad Prism 5 to check the qRT-PCR data. The fungal biomass in 168 hpi leaves of *P. purdomii* was also detected using trypan blue staining methods as outlined in Dang et al. (2013); these stained leaves were transferred into a chloral hydrate solution (2.5 g of chloral hydrate dissolved in 1 ml of distilled water) and boiled for 20 min to destain. The numbers and sizes of the developed fungal colonies of ΔTs_06_ and Ts_06_ in the tissues were compared.

**Table 2:** Primer sequences of small secreted proteins and the referred genes.

**Protein ID**	**Localization *in planta*^a^**	**Primer sequences (5^**′**^-3^**′**^) forward/reverse**	**Amplicon length (bp)**	**Annealing temperature**
124272	Nucleus and cytosol	ACCCTAGAAAGTCACCCAGC GTCGCACGTTTGAAATAGCCT	166	56
37347	Periphery of haustoria and plasmodesmata	ACTGGACCTGACTGCAACCGGATCTTGAGTAATGGTGGAAGG	169	55
124530	Nuclear and cytosolic bodies	TCTTTCTTTCTCTGGTCCATTTCTC CACGATGGCTTGACAGTCTAA	191	56
124256	Nucleus and cytosol	CCAACAGGTTTCAGTCAAGAGG GGTAGTGTGCTCCTTAGTCGT	195	56
123932	Nucleus and cytosol	TTGGATGATGTAGCAAGACTCGGTTATGCCTTTCAGCCGCT	105	55
Mlp-ITS	/	TGAGCGACTTTAATGTGACTCATGTAAATCAAAGTTGCCTTTGCG	123	54
Alpha-tubulin (*aTUB*)	/	ATCTGTAACGAACCTCCTGCTACCTCCTCCATACCTTCTCCAA	168	55
Elongation factor-1-alpha (*ELF1a*)	/	CGAGACTCCCAAATACTTCGTTGTTCACGAGTTTGACCATCCTT	167	55

a *As described in Petre et al. (2015)*.

### Transcriptome Sequencing and Differential Gene Expression Analysis

Ts_06_ urediniospores previously stored at −20°C were used to reproduce fresh urediniospores, which were marked CK20. ΔTs_06_ urediniospores stored at −20°C for 1 year were marked One20, and those stored for 2 years were marked Two20. Ts_06_ urediniospores previously stored at −80°C were used to reproduce fresh urediniospores, which were marked CK80. ΔTs_06_ urediniospores stored at −80°C for 1 year were marked One80, and those stored for 2 years were marked Two80. Two biological replicates were used for transcriptome analysis. Total RNA was extracted from 5 mg of urediniospores at the corresponding temperature and sequenced using BGISEQ-500 by Hua Da Biotechnological Co., Ltd. (Beijing, China). Clean reads were compared with the genome sequence of *M. larici-populina* (JGI; http://genome.jgi-psf.org/Mellp1/Mellp1.home.html) using hierarchical indexing for spliced alignment of transcripts (HISAT) software (Kim et al., 2015). The gene expression level of each sample was calculated using RSEM (RNA-Seq by Expectation Maximization), and the genes with a false discovery rate (FDR) ≤ 0.001 and an absolute value of the log2-fold change ≥ 1 were considered as differentially expressed genes (DEGs) (Li and Dewey, 2011). A Venn diagram, created using TBtools software, was used to illustrate the number of DEGs at the intersection of CK20 vs. One20, CK20 vs. Two20, CK80 vs. One80, and CK80 vs. Two80 (Chen et al., 2020). The expression clustering analysis of the intersecting DEGs was conducted using the heat map function in R software. According to the annotation results of Gene Ontology (GO) and the Kyoto Encyclopedia of Genes and Genomes (KEGG), an enrichment analysis was conducted on the intersecting DEGs using the phyper function in R software, and the *p* value was calculated and adjusted using FDR. Typically, functions with Q values ≤ 0.05 are regarded as significantly enriched. The intersecting DEGs were compared with the STRING database using DIAMOND (http://www.diamondsearch.org), and the relationship between interacting genes was obtained using homology and known proteins.

### Validation of Differentially Expressed Gene Expressions Using qRT-PCR

To verify the transcriptome data, genes differentially expressed in the mitotic and meiotic pathways and genes associated with “response to DNA damage stimuli” were selected for qRT-PCR verification. The alpha-tubulin (*aTUB*) and elongation factor-1-alpha (*ELF1a*) of *M. larici-populina* were used as reference genes (Hacquard et al., 2011). Primers were designed by software Beacon Designer 7.9 (Premier Biosoft International, USA, Table 3) according to the transcripts obtained in this experiment. The amplification reaction conditions followed Ye et al. (2018), and gene expressions were calculated using the 2^−ΔΔCt^ method (Livak and Schmittgen, 2001).

**Table 3:** Primer sequences of candidate genes and the referred genes.

**Gene symbol**	**Function annotation**	**Primer sequences (5^**′**^-3**0^′^**) forward/reverse**	**Amplicon length (bp)**	**Annealingtemperature**
MELLADRAFT_61177	Nucleic acid binding	AGTAGTCCTACGATCCATTCCAATACCCAAGTCCACAAGTTTCAC	145	55
MELLADRAFT_102486	Protein kinase activity	GGTCTTCTTCGGTTCTCAGGACAGTTGTAATCCACGCAGTAA	191	55
BGI_novel_G000167	Nucleic acid metabolic process	GTGCTGGACAACTAGGTGATAAGCCTCATCTGCTTCGTCATCTTG	222	55
MELLADRAFT_115157	DNA replication initiation	CGGCGTTGCTCACATCTTCACCTTCATTCCACCAGTCACA	102	56
MELLADRAFT_104525	Nucleic acid metabolic process	TCAAAGACATCAAAGCCTTCCTTGCCTTCTCGGAGTCAGTAAGTA	172	55
MELLADRAFT_36075	Nuclear division	TTACATTATCCACGGCTCTTCAAG TCTACCAGTCTACGCATTCTAC	154	56
MELLADRAFT_47540	Cellular response to stimulus	GGTTGATGGTGGTGTAAGGAACCGTAGGAATGTAGTTTGGGTTA	119	55
MELLADRAFT_33683	Response to stimulus, GTPase	CTCAGCAGGCGAGAAGGTCGTGACAAGCGTCTGGATT	104	55
MELLADRAFT_73904	adenylate cyclase	GCAAGCAATGACACGGATGAGCAAGCAATGACACGGATGA	156	56
MELLADRAFT_110829	Cyclin-dependent kinase	GGTATGGAGGCGTGTTATTAGGGGCTTGCTGAGGTTCTGAA	195	55
MELLADRAFT_112356	DNA damage stimulus	GATGCGAAGCCTGTGAAGAAGCGGTATAGACGAGATGATGATTG	155	56
BGI_novel_G000377	DNA damage stimulus	CTCGGTCTGATTGCGTTGTGACTGAGCCTCGTATGAATCTG	175	55
Alpha-tubulin (*aTUB*)	Reference genes	ATCTGTAACGAACCTCCTGCTACCTCCTCCATACCTTCTCCAA	168	55
Elongation factor-1-alpha (*ELF1a*)	Reference genes	CGAGACTCCCAAATACTTCGTTGTTCACGAGTTTGACCATCCTT	167	55

### Hydrogen Peroxide and Fatty Acid Detection

Six samples (CK20, One20, Two20, CK80, One80, and Two80) of 0.05 g of urediniospores were used to detect concentrations of H_2_O_2_ according to the protocol of Kit YX-W-A400 (Sinobest Bio Co., Ltd., Shanghai, China). In addition, 0.1 g of urediniospores from Two80 and 0.1 g from CK80 were selected to detect fatty acids using gas chromatography–mass spectrometry (GC-MS, QP2010; Shimadzu Corp., Kyoto, Japan) (Zhao et al., 2020). Each sample was biologically repeated three times, and the means of each detection were determined using Duncan's multiple range test (software SPSS 22.0, Chicago, IL, USA).

### Detection of Genotype Variation Between ΔTs_06_ and Ts_06_

We used the method in Virtudazo et al. (2001) to extract DNA from ΔTs_06_ and Ts_06_ urediniospores. Random amplified microsatellites (RAMs) were applied to screen for differences in whole genomic DNA between ΔTs_06_ and Ts_06_. The RAM primers were designed (Supplementary Table 1), and the PCR was carried out following the procedure outlined in Hantula et al. (1996). Amplification products were separated using electrophoresis in 2.0% agarose gels, and the DNA profiles of ΔTs_06_ and Ts_06_ were carefully compared. The occurrence of different DNA profiles manifests a different genotype at the allelic gene.

## Results

### Nuclei in the Life Cycle of ΔTs_06_

**Figure 2:**
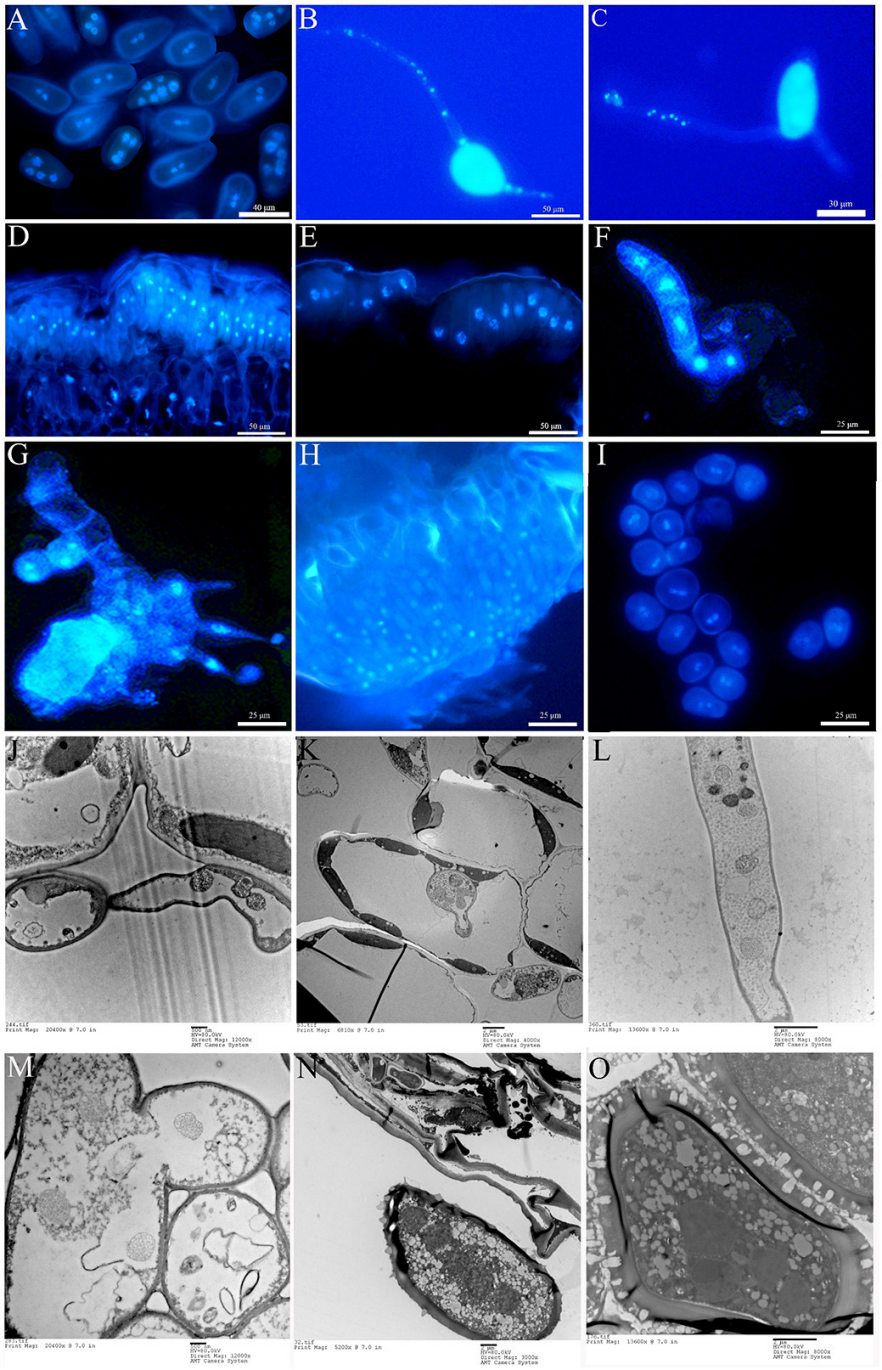
Nuclear observation of the life cycle of ΔTs_06_ in the. **(A)** DAPI staining of urediniospores. **(B,C)** Polykaryon germ tube. **(D)** Initial teliospores. **(E)** Mature teliospores. **(F)** Basidium. **(G)** Basidiospores. **(H)** Spermatia. **(I)** Aeciospores. **(J)** Polynuclear haustorial mother cell. **(K)** Polynucleated haustoria. **(L)** Polynucleated intercellular hyphae. **(M)** Fusing intercellular hyphae. **(N,O)** Polynuclear urediniospores.

During the uredial stage, both ΔTs_06_ urediniospores and their germ tubes contain four to six nuclei (Figures 2A–C). After successfully infecting *P. purdomii*, within 1 day, haustorial mother cells and haustoria developed and had two to three nuclei (Figures 2J,K). In addition, intercellular hyphae developed in the mesophyll tissue, and each somatic cell had three to five nuclei (Figure 2L). A few intercellular hyphae were fused together, and the nucleus flow along with them (Figure 2M). After approximately 6 dpi, intercellular hyphae finally developed into urediniospores that had four to five nuclei (Figures 2N,O), which took on about 20% of total fresh urediniospores during each of the five consecutive generations. However, Ts_06_ urediniospores, germ tubes, intercellular hyphae, haustorial mother cells, and haustoria are all typically dikaryotic (Supplementary Figure 1). During the telial stage, unmatured teliospores are initially binucleate (Figure 2D), and they fused into one nucleus after maturity (Figure 2E). After one time of meiosis, the basidium from the germination of teliospores is divided into four cells by three septa, and each cell has a nucleus (Figure 2F). The basidiospores are produced on the stigma of the promycelium. As the tip expands, one nucleus flows into it, and mitosis occurs. Finally, basidiospores with two nuclei are formed (Figure 2G). The basidiospores infect needles of *Larix* and produce spermogonia and mononuclear spermatia on the adaxial surface of the needles (Figure 2H). A week after the development of spermogonia, aecidium and binuclear aeciospores appeared on the abaxial surface of the needles (Figure 2I). There is no difference at nuclear number between the isolate Ts_06_ and ΔTs_06_ except the uredial stage and its infection structures (Supplementary Figure 1).

### Pathogenicity and Biomass of ΔTs_06_

**Figure 3:**
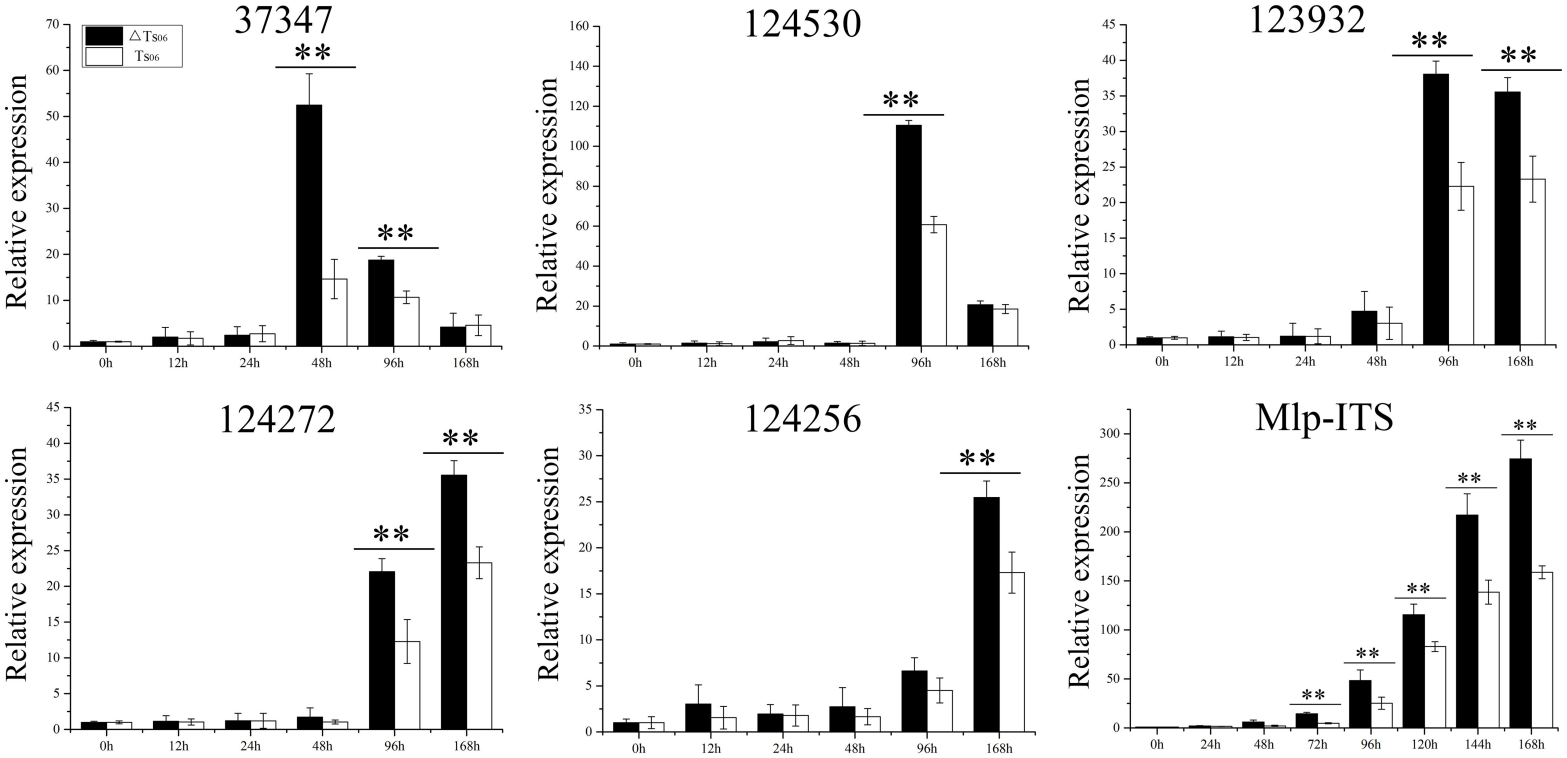
qRT-PCR of small secreted proteins (SSP) in ΔTs_06_ and Ts_06_ during *Populus purdomii* infection. Labeling error lines were obtained from three biological repetitions. Significant differences are indicated by **(*p* value <0.01).

Except for the incompatible host *P. deltoides*, ΔTs_06_ showed more compatibility and shortened the latent period by approximately 1 day compared with Ts_06_ on the other four hosts (Table 1). No significant difference was noted between ΔTs_06_ and Ts_06_ regarding uredinium diameter and height, but the density of ΔTs_06_ was significantly higher than that of Ts_06_ on the four hosts (*p* value <0.05) (Supplementary Figure 2), and the fungal colonies in ΔTs_06_-*P. purdomii* stained by trypan blue were much more in number than the colonies in Ts_06_-*P. purdomii* (Supplementary Figure 3). These results showed a bigger fungal biomass for ΔTs_06_ infection. The qRT-PCR of ITS also displayed fungal biomass of ΔTs_06_ and yielded higher than did Ts_06_, and both strains increased their biomass since 2 dpi and reached a maximum at 7 dpi (Figure 3). During 0~24 h after both ΔTs_06_ and Ts_06_ were inoculated with *P. purdomii*, no significant change was noted in the expression of SSP (Figure 3). However, at the 48th h, the expression level of protein 37347 was significantly higher in ΔTs_06_ than in Ts_06_, and ΔTs_06_ produced more haustoria than Ts_06_. At the 96th hour, the expression levels of four proteins (37347, 124530, 123932, and 124272) were significantly higher in ΔTs_06_ than in Ts_06_, and ΔTs_06_ produced more intercellular hyphae than Ts_06_. At the 168th h, the expression levels of three proteins (123932, 124272, and 124256) were significantly higher in ΔTs_06_ than in Ts_06_, and ΔTs_06_ produced more urediniospores than Ts_06_.

### Differentially Expressed Genes Associated With Temperature and Time

**Figure 4:**
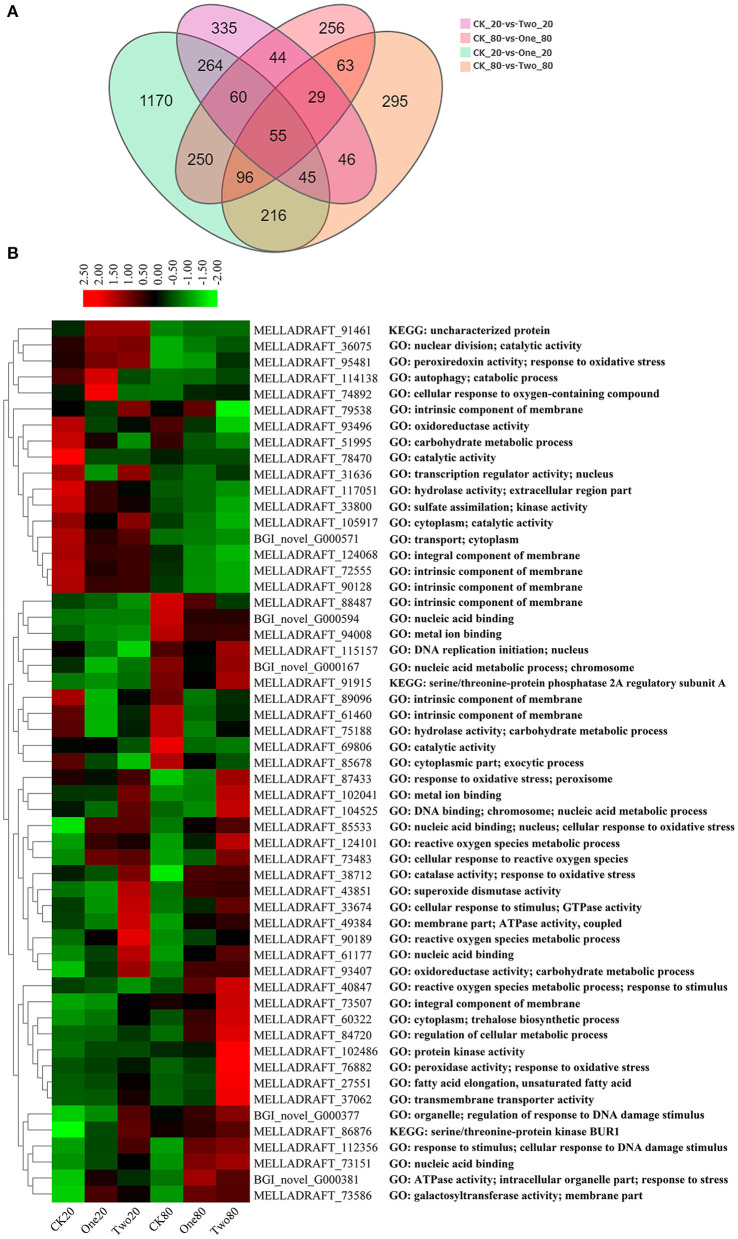
Differentially expressed genes (DEGs) at different cryopreservation temperatures. **(A)** Venn diagram showing the number of differentially expressed genes. **(B)** Cluster heat map of intersectional gene expressions.

An average of 42.5 M of clean reads per sample were collected (Supplementary Table 2). A total of 10,496 genes were detected, among which 9,869 genes were known and 627 new genes were predicted. Q30 (percentage of bases with quality > 30 in clean reads) for each sample was approximately 91%. The proportion of mapped reads ranged from 70.49% to 76.74%, and manifested RNA-Seq results were up to standard. There were 3,224 DEGs in the union of CK20 vs. One20, CK20 vs. Two20, CK80 vs. One80, and CK80 vs. Two80; and a total of 55 genes intersected (Figure 4A). Of these 55 genes, 30 were upregulated and 25 were downregulated (Figure 4B). The top five upregulated genes were related to “nucleic acid binding” (MELLADRAFT_61177 and MELLADRAFT_73151), “fatty acid elongation” (MELLADRAFT_27551), “transmembrane transporter activity” (MELLADRAFT_37062), and “reactive oxygen species metabolic process” (MELLADRAFT_90189). The top five downregulated genes were related to “catalytic activity” (MELLADRAFT_69806), the “intrinsic component of membrane” (MELLADRAFT_72555, MELLADRAFT_124068, and MELLADRAFT_90128), and the “oxidoreductase activity” (MELLADRAFT_93496).

**Figure 5:**
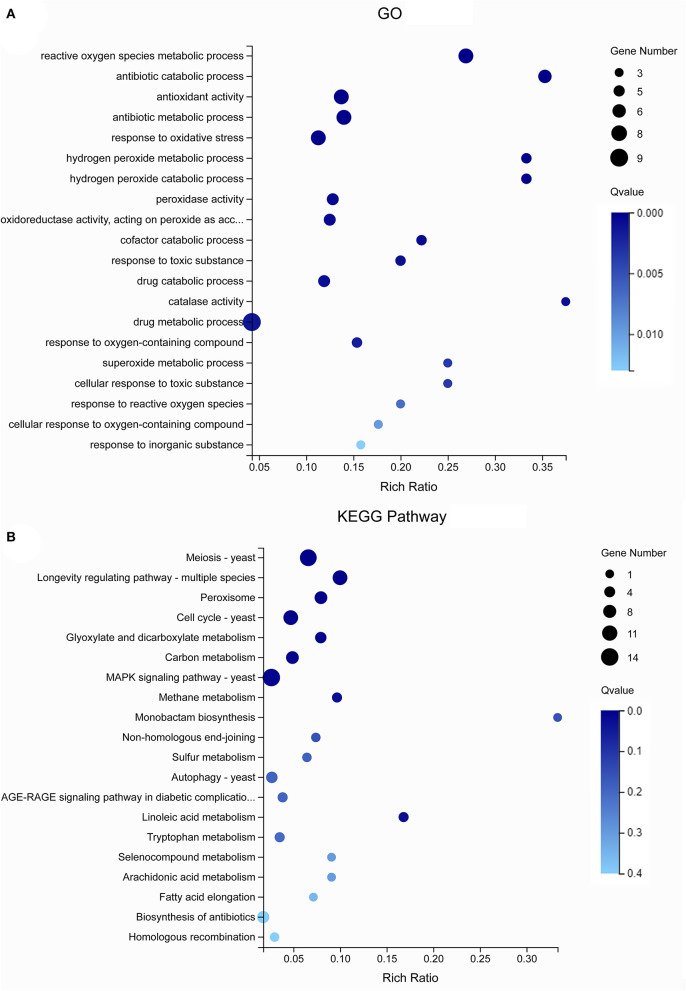
Gene Ontology (GO) and Kyoto Encyclopedia of Genes and Genomes (KEGG) analysis of differentially expressed genes (DEGs). **(A)** GO enrichment bubble diagram. **(B)** KEGG enrichment pathway diagram.

**Figure 6:**
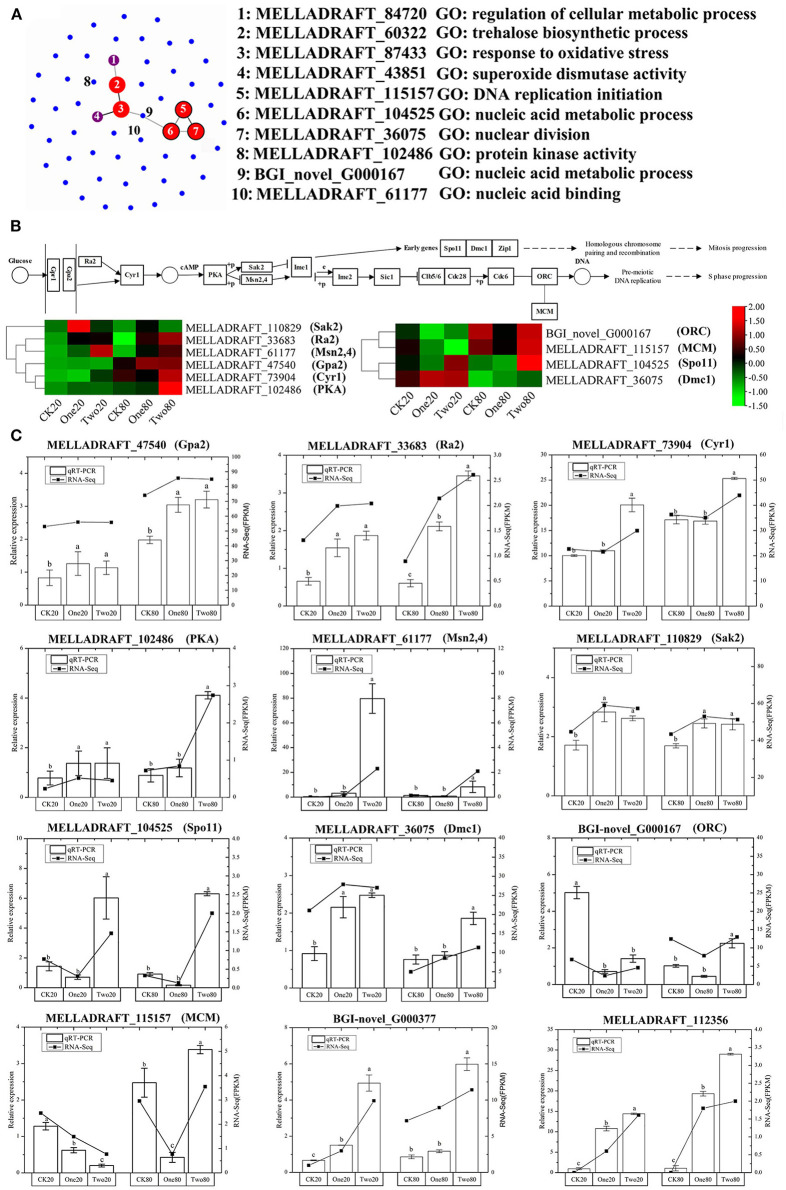
qRT-PCR verification of differentially expressed genes (DEGs). **(A)** Protein network diagram of DEGs. **(B)** Gene regulation pathway map of the G2 stage of mitosis and the S stage of meiosis. **(C)** qRT-PCR of DEGs. Note: Each point in the graph represents one gene, and the connecting lines between the two genes indicate that two genes interact. The size and color of the dots indicate the number of interactive connections, larger dots indicate that there are more connections, the color gradually changes from blue to red, and red indicates more connections. The range from green to red in **(B)** indicates the gene FPKM (fragments per kilobase of transcript per million fragments mapped) value. The error lines marked in **(C)** are obtained from three biological repetitions, and the significant differences are indicated by a, b, and c.

The GO analysis of these 55 genes revealed that the enriched items primarily included “drug metabolism” (GO: 0017144), “reactive oxygen species metabolism” (GO: 0072593), “antibiotic metabolism” (GO: 0016999), and “response to oxidative stress” (GO: 0006979) (Figure 5A). According to the KEGG analysis of the 55 genes, the primary enrichment pathways were the “MAPK signaling pathway” (ko04011), the “meiosis” (ko04013), the “longevity regulating pathway” (ko04213), the “cell cycle” (ko04111), and the “linoleic acid metabolism” (ko01212) (Figure 5B). Compared with the STRING database, the 55 genes formed a protein network diagram of 10 core genes (Figure 6A), which were annotated as “regulation of cellular metabolic process” (MELLADRAFT_84720), “trehalose biosynthetic process” (MELLADRAFT_60322), “response to oxidative stress” (MELLADRAFT_87433), “superoxide dismutase activity” (MELLADRAFT_43851), “DNA replication initiation” (MELLADRAFT_115157), “nucleic acid metabolic process” (MELLADRAFT_104525, BGI_novel_104525), “nuclear division” (MELLADRAFT_36075), “protein kinase activity” (MELLADRAFT_102486), and “nucleic acid binding” (MELLADRAFT_61177). Six of these 10 core genes are believed to be key genes in the mitotic and meiosis pathways (Figure 6B).

### Verification of Differentially Expressed Genes Using qRT-PCR

Ten genes in the mitotic and meiosis pathways and two genes of “responses to DNA damage stimulus” were selected for qRT-PCR verification (Figure 6C). The expression of qRT-PCR was consistent with FPKM (fragments per kilobase of transcript per million fragments mapped) analysis of the transcriptome. All three genes (MELLADRAFT_47540, MELLADRAFT_33683, and MELLADRAFT_73904) at the beginning of the nuclear division pathway were upregulated, which suggested that these genes responded to cryopreservation stimuli. The expressions of MELLADRAFT_102486 (PKA) in One20 and Two20 were significantly higher than those in CK20, and the expression in Two80 was significantly higher than that in One80 and CK80, indicating that the protein kinase was more active in 2-year preservation than in both the 1-year and the contrast. The expression level of MELLADRAFT_61177 (Msn2,4) in Two20 was significantly higher than the levels in CK20 and One20, and the expression in Two80 was also significantly higher than the levels in One80 and CK80, showing that as the temperature lowered and time increased, the expression became higher. The expression levels of MELLADRAFT_110829 (Sak2) in both Two20 and One20 were significantly higher than those in CK20, and the expressions in Two80 and One80 were also significantly higher than that in CK80, indicating that both Msn2,4 and Sak2 were regulated by the upstream gene PKA. The expression of BGI-novel_G000167 (ORC) related to nucleic acid metabolism in CK20 was significantly higher than the levels in One20 and Two20, but the expression in Two80 was significantly higher than the levels in One80 and CK80. The expression of MELLADRAFT_115157 (MCM) in CK20 was significantly higher than the levels in One20 and Two20, which was consistent with its upstream gene (ORC) expression, but its expression in Two80 was significantly higher than the levels in CK80 and One80. Since the ORC and MCM genes are both upstream meiotic genes, cryopreservation at −80°C for 2 years (as in CK20) was determined to accelerate meiotic progression. The expression level of MELLADRAFT_104525 (Spo11) in Two20 was significantly higher than the levels in CK20 and One20, and its expression in Two80 was significantly higher than the levels in CK80 and One80, suggesting an upward trend in DNA replication as cryopreservation time increased. The expression levels of MELLADRAFT_36075 (DMC1) in Two20 and One20 were significantly higher than those in CK20, and the expression in Two 80 was significantly higher than the levels in One80 and CK80, exhibiting an upward trend of nuclear division similar to its upstream gene Spo11. The expression levels of DMC1 and Spo11 accelerated mitosis progression, and a longer cryopreservation time enabled higher activity in nuclear mitosis. Two80 displayed strong dynamics in both mitosis and meiotic progression. Another two genes of “responses to DNA damage stimulus” (BGI-novel_G000377 and MELLADRAFT_112356) were upregulated, which hinted that the DNA damage of urediniospores became more severe as cryopreservation time increased (Figure 6C).

### H_2_O_2_ Concentration and Fatty Acid Content in Urediniospores

**Figure 7:**
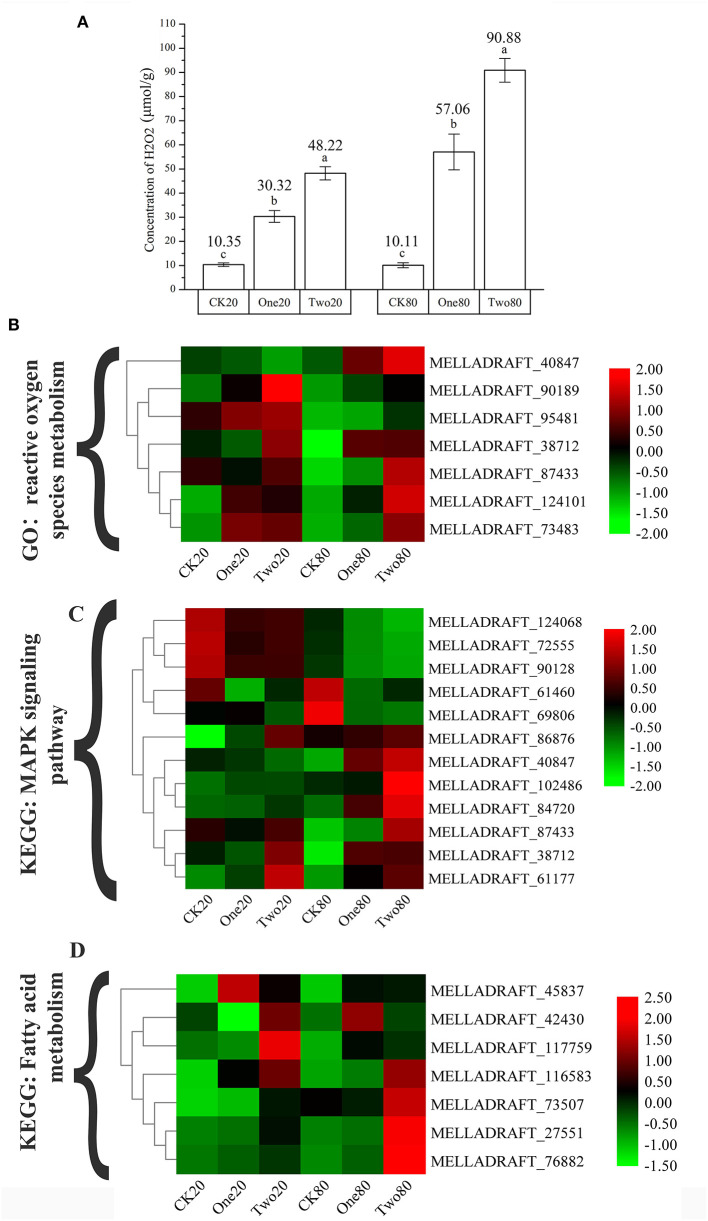
Determination of the concentrations of H_2_O_2_ in different samples and the cluster heat map of different gene expressions. **(A)** Determination of the concentrations of H_2_O_2_. **(B)** The genes of “reactive oxygen species metabolic process.” **(C)** The genes of “MAPK signaling pathway.” **(D)** The genes of “fatty acid metabolism.” Different letters mean significant differences at the 0.05 level.

The concentrations of H_2_O_2_ in Two20 and Two80 were significantly higher than those in One20 and One80, while the concentrations of H_2_O_2_ in One20 and One80 were significantly higher than those in CK20 and CK80 (Figure 7A). This result indicated that “reactive oxygen species” (ROS) increased as cryopreservation time increased, and the result agreed with the transcriptome results (Figure 7B) that longer preservation time increased the superoxide content. Seven fatty acid components were massive in the urediniospores of both ΔTs_06_ and Ts_06_, six of which (hexadecanoic acid, 9,12-octadecadienoic acid, and 9,12,15-octadecatrienoic acid, etc.) were found in greater quantities in ΔTs_06_ than in Ts_06_ (Supplementary Table 3).

### Comparison of Genotypes of ΔTs_06_ and Ts_06_

The DNA profile from RAM amplification showed that the two strains had different fragments, although only four primers were employed in this study. However, a difference in genomic DNA was detected. This result indicated that genotype variation occurred between the two strains during cryopreservation (Supplementary Figure 4).

## Discussion

Nuclear mutation is a main way to form multinucleation phenomena (Roper et al., 2011). The leading cause of nuclear mutation is abnormal mitosis (Yoshida et al., 2007; Wang et al., 2013). Apopa et al. (2009) reported that the presence of excessive ROS in cells resulted in abnormal mitosis and multinuclear cells. This study identified 55 DEGs at the intersection of CK20 vs. One20, CK20 vs. Two20, CK80 vs. One80, and CK80 vs. Two80. According to the GO enrichment analysis of these 55 genes, “reactive oxygen species metabolism” and “response to oxidative stress” are two items that possess many enriched genes, and each of them is enriched with seven genes (Figure 5A). The functional annotation “reactive oxygen species” (MELLADRAFT_90189, MELLADRAFT_124101, MELLADRAFT_73483, and MELLADRAFT_87433) was significantly upregulated in One20, Two20, One80, and Two80 (Figure 7B). Correspondingly, the concentrations of H_2_O_2_ in urediniospores of both Two20 and Two80 were significantly higher than those in CK20 and CK80 (Figure 7A). The expression levels of the two genes of “responses to DNA damage stimulus” (BGI-novel_G000377 and MELLADRAFT_112356) were significantly higher in One20, Two20, One80, and Two80 than those in CK20 and CK80 (Figure 6C). These results indicated that Ts_06_ stored at −80°C increased the expression of ROS in urediniospores, which resulted in damage to nuclear DNA and eventually led to abnormal nuclear mitosis and the formation of polynuclear spores. Meiosis is believed not to occur during the uredinia stage in the life cycle, but synapses can be found before haploid karyon fusion in *M. larici-populina*. In this study, the S-phase progression genes, ORC and MCM, which may also be involved in the formation of polykaryon, were upregulated at −80°C. The qRT-PCR analysis of 10 genes in the mitotic and meiotic pathways showed that the expression levels of three genes (MELLADRAFT_47540, MELLADRAFT_33683, and MELLADRAFT_73904) were upregulated, suggesting that these genes responded to environmental stimuli. Except for these genes, expressions of the protein kinase activity gene MELLADRAFT_102486 (PKA) and the nucleic acid metabolism gene MELLADRAFT_61177 (Msn2,4) also increased, and the downstream genes related to mitosis and S-phase meiotic division were both activated. For example, MELLADRAFT_36075 (Dmc1), MELLADRAFT_104525 (Spo11), BGI-novel_G000167 (ORC), and MELLADRAFT_115157 (MCM) exhibited upward trends of expression at −80°C as cryopreservation time increased. Abnormal mitosis and abnormal S-phase progression of urediniospore nuclei in ΔTs_06_ were believed to cause polynuclear phenomena in this study.

Mitogen-activated protein kinases (MAPKs) are important factors in eukaryotic signaling networks (Bögre et al., 1999). The MAPK signal cascade is crucial for sensing environmental stimuli on the cell surface and transmitting these signals to the nucleus to regulate gene expression (Qi and Elion, 2005). Most of the 55 KEGG-annotated DEGs were in the MAPK signaling pathway. Of these 55 genes, MELLADRAFT_84720, MELLADRAFT_87433, MELLADRAFT_102486, and MELLADRAFT_61177 were also found in the networks of interactive proteins (Figures 6A, 7C). Furthermore, MELLADRAFT_102486 and MELLADRAFT_61177 are upstream genes in mitosis and meiosis, and they both showed an upward expression trend during cryopreservation, which suggested that the MAPK signaling pathway played an important regulatory role in abnormal nuclear division.

Transcriptome sequencing revealed that genes associated with “fatty acid metabolism” (MELLADRAFT_117759, MELLADRAFT_73507, MELLADRAFT_45837, and MELLADRAFT_27551) and “trehalose biosynthesis process” (MELLADRAFT_60322) were significantly upregulated (Figure 7D). Trehalose is widely noted in bacteria, fungi, and plants (Avonce et al., 2006). In fungi, trehalose is often used as a carbon storage source and responds adaptively to various pressure conditions (Thevelein, 1984; Gancedo and Flores, 2004), including dehydration, oxidative stress, heat treatment, cold treatment, and freezing stress (Sasano et al., 2012; Zakharova et al., 2012). Notably, fatty acids affect the biochemical activity, transfer process and stimulation of cells, and participate in several physiological processes, including lipid metabolism, cell recognition, immune response, and cold adaptation (Grammatikos et al., 1994; Jump et al., 1996). The metabolism of fatty acids and trehalose in urediniospores increased during the cryopreservation (Supplementary Table 3); this increase in metabolism hinted an improvement of urediniospores to resist damage due to low temperatures.

Many studies have shown that avirulence genes or effectors encoded by pathogenicity-related genes are secreted proteins (Zhang et al., 2020), and secreted proteins are assumed to be key molecules for pathogenicity (Petre et al., 2014). For example, AvrL567 and AvrP4 are secreted proteins in *M. lini* (Petre et al., 2014). Avirulence gene is one of the important SSP genes, which often appears in the form of a gene family and plays a role during the infection (Dodds et al., 2004). In this study, the expressions levels of five SSPs homologous with avirulence genes of *M. lini* were used to compare the pathogenicity of ΔTs_06_ and Ts_06_ during the infection. The levels were significantly higher in ΔTs_06_ than in Ts_06_ at 48, 96, and 168 h probably because ΔTs_06_ produced much more haustoria, intercellular hyphae, and urediniospores than Ts_06_ did. In addition, the higher urediniospore density and shorter latent period of ΔTs_06_ on the compatible host *P. purdomii* also validated these SSP expressions, and ΔTs_06_ displayed more compatibility and more pathogeneticity with *P. purdomii*. Furthermore, the RAM screen based on genomic DNA determined that the genotype of ΔTs_06_ was different from that of the isolate Ts_06_. Cryopreservation changed both the phenotype and genotype of isolate Ts_06_.

The nuclear behavior in the life cycle of rust fungi is of great significance for understanding individual inheritance, interspecific evolutionary, and intraspecific pathogenicity variation of rust fungi. Except for Uredinales imperfecti, all rust fungi have telial stages in their life cycle. Teliospores are diploid cells that need two haploid nuclei to fuse before they can germinate and start the life cycle. Germination of teliospores is the most important event in the life cycle of rust fungi because both karyogamy and meiosis occur at this stage. In fact, meiosis occurs before the germination of teliospores. After karyogamy, diploid cells quickly complete homologous chromosome pairing, and the synaptonemal complex is produced in the dormant period (Mims and Richardson, 2005). The genes related to prophase I, such as the Spo11 protein gene in the leptotene stage, the tetrad-forming kinase Hop1 gene in the zygotene stage, Rad51 and Mnd1 in the pachytene stage, and karyokinesis genes were significantly upregulated (Hacquard et al., 2013). However, due to the differences in the time and position of karyogamy among different rusts, the shape of basidium (promycelium) produced by teliospores, the cell position during meiosis, the number of basidiospores, the times of re-germination of basidiospores, and the nuclear status are all obviously different (Shimomura et al., 2012), which indicate a diversity of life cycles. Furthermore, Jackson (1935) described seven types of nuclear behaviors of microcyclic rust, Petersen (1974) described six types, Hiratsuka and Sato (1982) described eight types, and Ono (2002) described 10 types and 21 subtypes of microcyclic rust. In this study, the telial and basidial stages of polykaryon ΔTs_06_ were not different than those in a typical macrocyclic *M. larici-populina*; how polykaryotic somatic hyphae develop into karyogamy and how meiosis ultimately occurs are still not well understood.

Cummins and Hiratsuka (1983) thought that heteroecious macrocyclic rust often shortened the life cycle and formed the corresponding species. In a disturbed ecosystem, macrocyclic rust may lose the opportunity and the ability to complete heterosexual mating and evolve from the unstable parent population. This repeated evolution makes up for the danger of macrocyclic rust dying out in harsh evolutionary environments, and thus, many branches of microcyclic rust are produced and distributed in different niches (Hennen and Buritica, 1980). For example, *Peridermium yamabense* is a synonym for *Endocronartium yamabense* because it cannot form real teliospores and is the asexual type of *Cronartium ribicola* (Imazu et al., 1991). Zemodeme I of *E. harknessii* (≡*P. harknessii*) and *C. quercuum* are highly similar. Zemodeme II originates from zemodeme I after karyogamy, or the latter is a haploid from the former (Vogler et al., 1997). Another example is the leaf rust *Rubus* sp., which has three species. One is the demicyclic rust *Gymnoconia peckiana*, which forms vegetative aeciospores on the leaves and germinates to produce binuclear germ tubes and an appressorium. The other two species are endocyclic rust *G. nitens*, which can produce aeciospore-type teliospores on leaves. One of the *G. nitens* species germinates to form a two-cell basidium and produces two haploid basidiospores, whereas the other one forms four-cell basidiospores and grows four basidiospores. In four-cell basidiospores, two nuclei in aeciospore-type teliospores do not undergo karyogamy and meiosis but rather is formed by direct division of the binucleate form (Mims et al., 2007). The multinucleation phenomenon produced by urediniospores stored during cryopreservation is an important basis for the diverse evolution of the life cycle of *M. larici-populina*, which is of great significance for improving the adaptability of rust fungi to low-temperature environments, genetic variation, and host selection.

## Conclusion

Cryopreservation can change both the phenotype and genotype of isolate Ts_06_. The multinuclear isolate ΔTs_06_ has stronger host compatibility, pathogenicity, and environmental adaptability than Ts_06_ has. Transcriptomics suggested that the fatty acids metabolism in cryopreserved urediniospores increased, which improved the ability of urediniospores to resist damage caused by ultralow temperatures. Cryopreservation triggered the MAPK signaling pathway and led to an increase in reactive oxygen metabolism in urediniospores; then, nuclear DNA damage and abnormal mitosis and meiosis processes facilitated the formation of polykaryotic urediniospores. In the continuous subculture, the proportion of polykaryotic urediniospores of ΔTs_06_ exhibited no significant change. At the 0, I, III, and IV stages in the life cycle, the number of nuclei is not different between ΔTs_06_ and Ts_06_. The results indicated that this mutant strain had a certain stability and environmental adaptability. However, how the polykaryon strain completes binucleation and undergoes meiosis remains unclear.

## Data Availability Statement

The datasets generated for this study can be found in the NCBI Sequence Read Archive (PRJNA673784, https://www.ncbi.nlm.nih.gov/sra).

## Author Contributions

ZY and ZC conceived and planned the study. WZ performed the field investigation and characterization experiments and analyzed the data. ZY and WZ contributed key ideas, analyzed the data, and wrote the manuscript. ZP and SP assisted in the data analysis. All authors contributed to the article and approved the submitted version.

## Conflict of Interest

The authors declare that the research was conducted in the absence of any commercial or financial relationships that could be construed as a potential conflict of interest.
