# Supplementary material for: Multinuclei Occurred Under Cryopreservation and Enhanced the Pathogenicity of Melampsora larici-populina
Source: Front Microbiol. 2021 Jun 25;12:650902. doi: 10.3389/fmicb.2021.650902 (PMC8270653; doi:10.3389/fmicb.2021.650902)
Supplement: Supplementary file 1 [file Data_Sheet_1.docx]

Supplementary Material

# Supplementary Figures and Tables

## Supplementary Figures


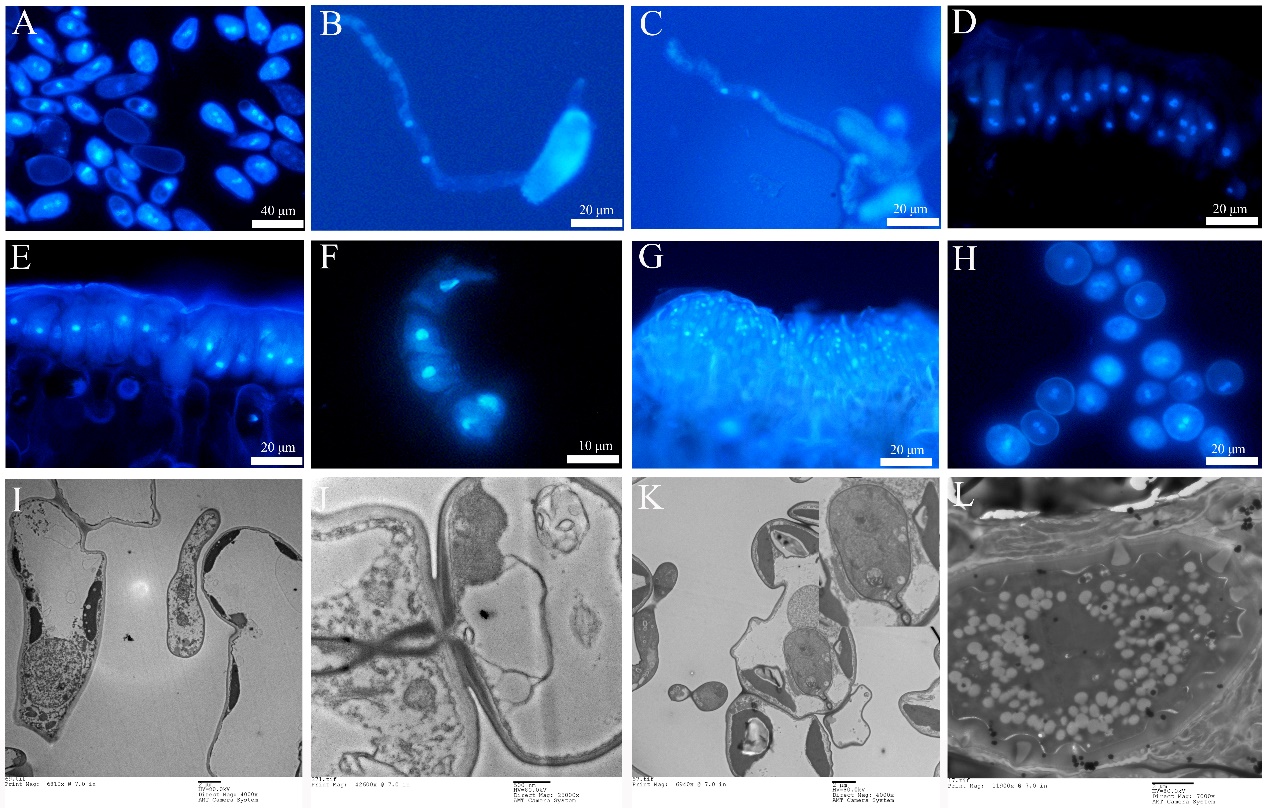


Supplemental Figure S1. Nuclear behavior in the Lifecycle of Ts_06._ **(A)** Dikaryotic urediniospores, **(B, C)** Dikaryotic germ tube, **(D)** Dikaryotic teliospores (unmatured), **(E)** Monokaryotic teliospores (matured), **(F)** Basidia with 1 nucleus in each cell, **(G)** Monokaryotic pycniospores, **(H)** Dikaryotic aciospores, **(I)** Dikaryotic intercellular hyphae, **(J)** Dikaryotic haustoria mother cell, **(K)** Dikaryotic haustoria, **(L)** Dikaryotic urediniospore.


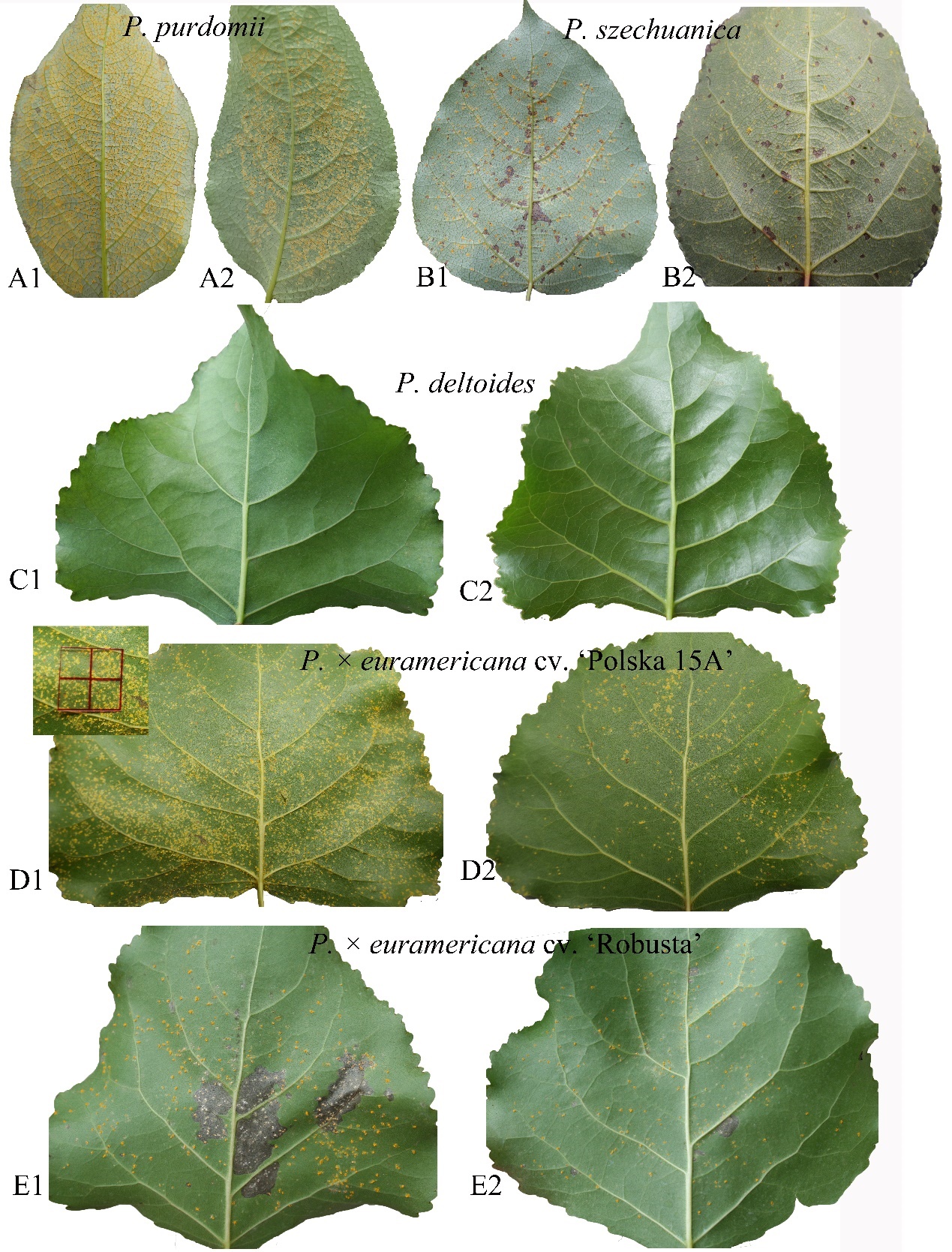


Supplemental Figure S2. The phenotypes of poplar leaves inoculated with △Ts_06_ and Ts_06_ at day 7. **A1**-**E1:** Poplar leaves inoculated with △Ts_06_, **A2-E2**: Poplar leaves inoculated with Ts_06_.


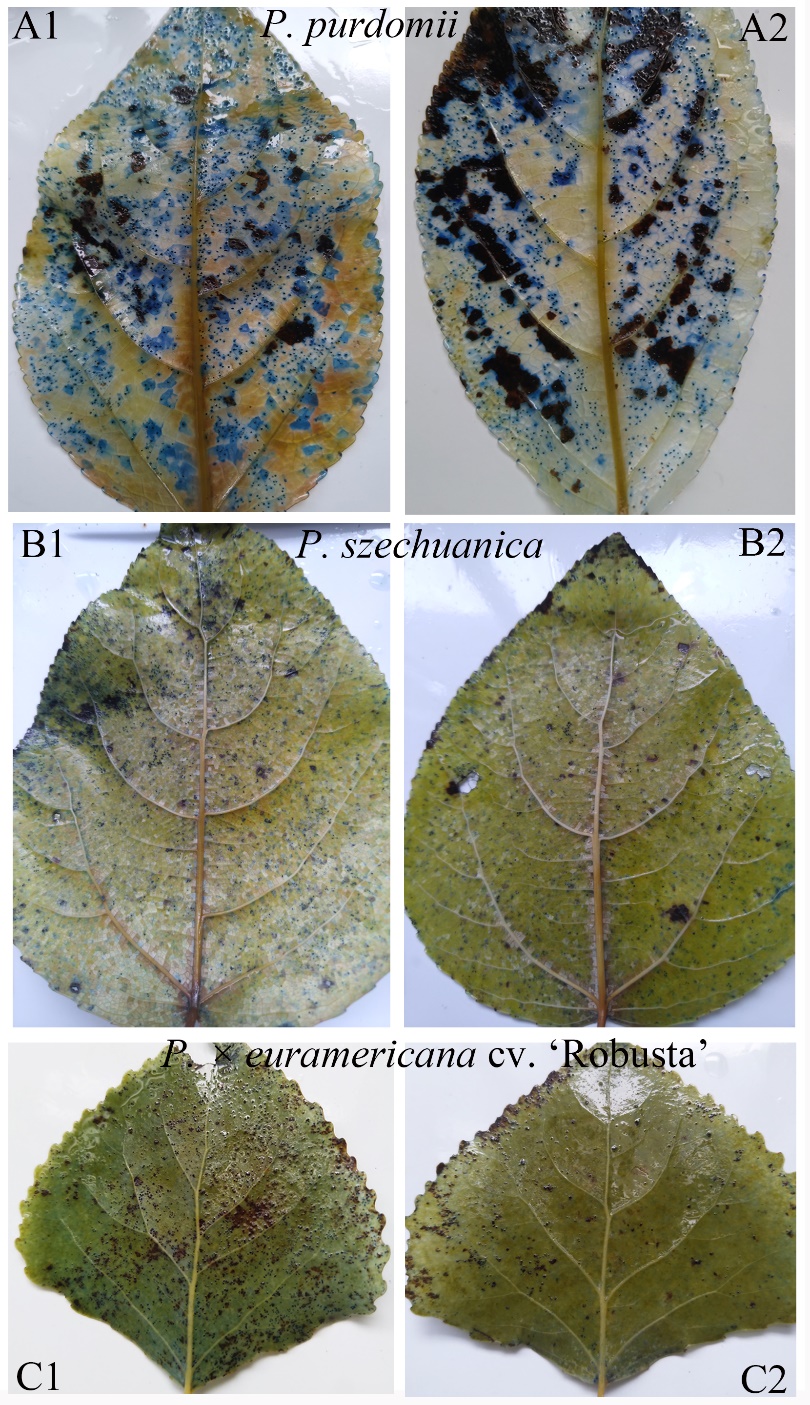


Supplemental Figure S3. The phenotypes of the trypan blue staining in poplar leaves induced by △Ts_06_ and Ts_06_ at day 7 and observation of fungi biomass. A1-C1: Poplar leaves infected with △Ts_06_, A2-C2: Poplar leaves infected with Ts_06_.


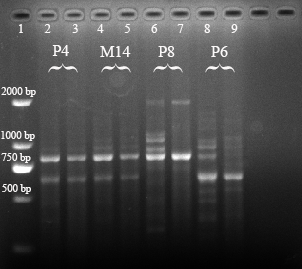


Supplemental Figure S4. Electrophoresis map of PCR products with RAMs. Note: Lane 1 represent the marker was DL2000, Lane 2, 4, 6 and 8 represents △Ts_06_, Lane 3, 5, 7 and 9 represents Ts_06_.

## Supplementary Tables

Supplemental Table S1. The tested RAMs primers and anneal temperature.

| **Primer** | **Primer sequence** | **Anneal temperature** |
| --- | --- | --- |
| P4 | 5’ GCG(CGA)_5_ 3’ | 54℃ |
| M14 | 5’ GAGGGTGGCGGTTCT 3’ | 48℃ |
| P8 | 5’ CCT(TCG)_5_ 3’ | 54℃ |
| P6 | 5’ ACT(CGA)_5_ 3’ | 52℃ |

Supplemental Table S2. The results of sequenced reads mapping to the reference genome.

| Samples | Total Clean Reads (M) | Total Clean Bases (Gb) | Q30 (%) | Total Gene | Mapping rate (%) |
| --- | --- | --- | --- | --- | --- |
| CK_20 | 42.22 | 6.33 | 91.65 | 9593 | 74.65 |
| CK_20 | 42.84 | 6.51 | 91.84 | 9628 | 74.05 |
| CK_80 | 41.97 | 6.30 | 89.75 | 8772 | 71.11 |
| CK_80 | 42.75 | 6.62 | 90.22 | 8851 | 71.90 |
| One_20 | 42.38 | 6.36 | 91.82 | 9535 | 76.74 |
| One_20 | 41.91 | 6.12 | 92.37 | 9420 | 76.34 |
| One_80 | 43.44 | 6.52 | 89.52 | 8752 | 71.65 |
| One_80 | 42.56 | 6.03 | 90.44 | 8697 | 72.70 |
| Two_20 | 42.60 | 6.39 | 92.07 | 9508 | 74.91 |
| Two_20 | 43.03 | 6.08 | 93.22 | 9845 | 72.53 |
| Two_80 | 43.25 | 6.49 | 89.83 | 8861 | 70.49 |
| Two_80 | 41.62 | 6.19 | 91.78 | 8646 | 71.83 |

Supplemental Table S3. The percentage of seven fatty acid components in the total fatty acid content of the sample.

| **Fatty acid component** | **For short** | **Two80 (%)** | **CK80 (%)** |
| --- | --- | --- | --- |
| Hexadecanoic acid, methyl ester | C16:0 | 16.00 ± 0.78^a^ | 12.80 ± 0.65^b^ |
| 9,12-Octadecadienoic acid (Z, Z)-, methyl ester | C18:2 | 16.61 ± 1.30^a^ | 15.30 ± 0.39 ^b^ |
| 9,12,15-Octadecatrienoic acid, methyl ester, (Z, Z, Z)- | C18:3 | 20.64 ± 1.67 ^a^ | 19.43 ± 0.86^b^ |
| Methyl stearate | C18:0 | 4.64 ± 0.12^a^ | 4.49 ± 0.4^a^ |
| 9-Octadecenoic acid (Z)-, methyl ester | C18:1 | 0.95 ± 0.17^a^ | 0.79 ± 0.08^b^ |
| Oxiraneoctanoic acid, 3-octyl-, methyl ester | C18:0 | 14.78 ± 1.27^a^ | 13.46 ± 2.36^b^ |
| Octadecanoic acid, 10-oxo-, methyl ester | C18:0 | 26.28 ± 1.40^a^ | 25.75 ± 2.47^b^ |
